# Infrared Spectral Descriptors for Reaction Yield Prediction: Toward Redefining Experimental Spaces

**DOI:** 10.1002/minf.70019

**Published:** 2026-02-12

**Authors:** Yuya Endo, Hiromasa Kaneko

**Affiliations:** ^1^ Department of Applied Chemistry School of Science and Technology Meiji University Kawasaki Japan

**Keywords:** descriptor, experimental design, infrared spectra, infrared spectroscopy, phosphine ligands, yield prediction

## Abstract

Yield prediction in catalytic reactions is essential for improving chemical process efficiency and product quality. Ligands significantly influence reactivity and selectivity, highlighting the need for descriptors that accurately capture their structural and electronic properties. In this study, we focus on infrared (IR) spectra, which reflects molecular vibrational modes, and propose novel descriptors based on wavenumber information. We evaluated the predictive performance of these descriptors using two datasets: direct Pd‐catalyzed arylation and Suzuki–Miyaura coupling reactions. The wavenumber‐based IR descriptors outperformed conventional molecular descriptors and structural fingerprints (one‐hot encoding, Mordred, MACCS, Morgan fingerprint, RDKit, and density functional theory). Notably, descriptors limited to the fingerprint region (0–1700 cm^−1^) effectively captured key molecular features, contributing to both high prediction accuracy and improved chemical interpretability. Our results indicate that IR‐based descriptors can achieve strong generalization performance even with small datasets. This approach offers a promising strategy for redefining reaction condition spaces and enhancing the interpretability of predictive models, thereby supporting more informed experimental design.

## Graphical abstract

Wavenumber‐based IR descriptors improve yield prediction in catalytic reactions by capturing ligand structure and electronics. They outperform one‐hot encoding, RDKit, Mordred, MACCS, Morgan, and density functional theory descriptor, offering a robust tool for guiding ligand selection and optimizing experimental conditions.

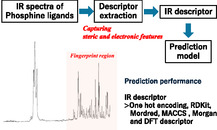

## 1 Introduction

In the fields of chemical and fine chemical synthesis, considerable time and cost are routinely invested to efficiently produce target compounds with the desired quality. Achieving high yields is particularly important, as it directly simplifies purification processes and contributes to improved process efficiency and product quality. In catalytic reactions, ligand selection plays a central role, as it directly affects both reactivity and selectivity, making it a key factor in reaction condition optimization.

Recent advances in high‐throughput experimentation (HTE) and Bayesian optimization have significantly improved the efficiency of exploration within predefined reaction condition spaces, and these methods are becoming standard in industrial and academic settings [1, 2, 3, 4]. However, the definition of reaction spaces still relies heavily on expert intuition, and due to the complex and multifactorial nature of chemical reactions, optimization workflows may not always yield satisfactory results. Therefore, there is a growing need to construct modeling frameworks that accurately capture the relationship between reaction conditions *x* and yield *y* as *y* = *f*(*x*), enabling effective redefinition of reaction spaces based on experimental data.

Yield prediction has evolved from early quantitative structure–activity relationship models [5] and Hammett equations [6] to modern approaches based on machine learning (ML) and deep learning (DL). Quantitative modeling of yield and selectivity requires simultaneous consideration of multiple factors, such as reagents, solvents, additives, and ligands, making it a multidimensional problem [7]. To handle such complexity, the design of descriptors that appropriately represent the chemical properties of each factor is essential.

Reghvan et al. [8] and Voinarovska et al. [9] categorized yield prediction models into local and global approaches based on dataset scale. Local approaches aim to extract maximum insight from relatively small experimental datasets, while global approaches integrate multiple reported datasets or databases to build generalizable models using DL techniques.

In local approach studies, molecular descriptors (e.g., Mordred [10]), structural fingerprints (e.g., MACCS [11], Morgan [12]), and density functional theory (DFT) ‐based descriptors have been widely used [13]. Wu et al. [14] developed a linear regression model for Ni‐catalyzed Suzuki–Miyaura coupling (SMC) reactions using electronic and steric features of ligands (e.g., Tolman cone angle, %Buried Volume, and minimum electrostatic potential), achieving both yield prediction and mechanistic insight. Although the ligand set was limited, the importance of capturing combined electronic and structural features was demonstrated. Conversely, Ahneman et al. reported high predictive accuracy using a random forest model with DFT descriptors on HTE data for Buchwald–Hartwig reactions. However, Chuang et al. [15] later showed that random features could yield comparable performance, raising concerns about the robustness of DFT‐based descriptors. Similarly, Żurański et al. [16] also found that the utility of DFT descriptors varies significantly across reaction systems, suggesting that descriptor selection may strongly depend on the specific reaction context.

Ligands directly influence reactivity, selectivity, and suppression of side reactions in catalytic processes. Accurately representing their electronic and steric properties is therefore critical for improving the performance of yield prediction models. Existing descriptors—including those derived from DFT—often struggle to capture subtle variations among ligands and may lack generalizability across different reaction systems. While DFT calculations can provide optimized structures from which steric parameters, such as %Buried Volume and Tolman cone angle, are derived, these descriptors are typically based on a single static conformation under idealized conditions. They do not account for dynamic effects, solvent interactions, or context‐dependent steric incompatibilities that arise in real catalytic environments. For example, Buchwald‐type phosphine ligands (e.g., JohnPhos, XPhos), which are highly effective in Pd‐catalyzed reactions, often fail in Ni‐catalyzed systems due to steric incompatibilities, such as excessive %Buried Volume, as demonstrated [14]. However, recent studies indicate that this effect is context dependent, underscoring the need for more comprehensive and physically meaningful representations [17].

To address these challenges, this study focuses on the use of infrared (IR) spectra. Unlike conventional DFT descriptors that primarily capture electronic properties, IR spectra encode vibrational modes that are inherently sensitive to both electronic distribution and three‐dimensional molecular geometry, including steric hindrance and intramolecular interactions. IR spectra provide molecule‐specific vibrational information that simultaneously reflects electronic and structural features, making them a promising source for ligand descriptor design [18, 19]. They can capture functional group types, intramolecular interactions, steric hindrance, and electron density distribution. IR data are accessible through experimental measurements, DFT calculations, and increasingly through ML‐based generation [20] and interpretation [21, 22], offering advantages in both availability and interpretability.

Despite the importance of ligand representation, current yield prediction models lack systematic approaches for ligand‐specific descriptor design. Many models rely on one‐hot encoding (OHE) or general‐purpose molecular descriptors and fingerprints, which are insufficient for capturing subtle reactivity differences. While conventional DFT descriptors face limitations in generalizability, IR spectra inherently encode both electronic and structural information, providing an opportunity to improve predictive accuracy and chemical interpretability. Given this background, there is a clear need for new descriptor designs that incorporate both electronic and structural information, such as that provided by IR spectra. By extracting features that reflect ligand reactivity and integrating them into yield prediction models, it is expected that both predictive accuracy and chemical interpretability can be improved [7].

In this study, we aim to enhance the accuracy of yield prediction models by designing descriptors based on IR spectra of ligands, which inherently encode both electronic and structural properties. We hypothesize that IR‐based descriptors can more precisely capture ligand reactivity compared to conventional fingerprints, and that they can achieve high predictive performance and interpretability even in small‐scale datasets, such as those obtained from HTE.

## 2 Methods

### 2.1 Descriptor Design Strategy

This study aims to design chemical descriptors based on IR spectra to simultaneously capture the electronic and structural properties of ligands. IR spectra contain information derived from molecular vibrational modes and comprehensively reflect functional group types, intramolecular interactions, steric hindrance, and electron density distribution. To ensure consistency and reproducibility of the descriptors, theoretical spectra obtained via DFT calculations were used instead of experimental spectra from external databases. Theoretical spectra provide idealized peak profiles without overlapping, allowing for clearer interpretation. Although computed from DFT‐optimized structures, IR spectra differ fundamentally from conventional DFT‐derived descriptors by encoding vibrational modes that reflect both electronic distribution and three‐dimensional geometry, offering richer and more dynamic information than static steric parameters.

### 2.2 Acquisition of Infrared Spectra

For a set of target compounds (*n* samples), molecular structure files in SDF format were prepared, and DFT calculations were performed using Gaussian software [23]. The 6‐31G(d, p) basis set was employed to obtain IR spectra for each compound. The resulting spectra were sorted by wavenumber, and two spectral regions were examined: 0–4000 and 0–1700 cm^−1^.

### 2.3 Spectral Clustering and Feature Extraction

*K*‐means clustering was applied to the wavenumber domain of each spectrum, dividing the data into 5–20 clusters. The number of clusters was fixed for each pattern, and descriptors were generated for all cluster configurations for subsequent model evaluation.

Two types of descriptors were constructed for each cluster:

• Intensity‐based IR descriptor (IntIR): Spectral intensity values were normalized to a range from 0 to 100. The highest intensity peak within each cluster was extracted as the representative feature. If no peak was present in a cluster, a value of 0 was assigned.
• Wavenumber‐based IR descriptor (WaveIR): Each cluster was defined by its wavenumber range (minimum to maximum), and the wavenumber of the highest intensity peak within that range was extracted as the representative feature. If no peak was present, the minimum wavenumber of the cluster range was used.

Descriptor names were abbreviated according to the spectral region, such as “IntIR040” and “WaveIR017,” where “040” and “017” denote the 0–4000 and 0–1700 cm^−1^ regions, respectively.

### 2.4 Descriptor Generation Workflow

i. Prepare molecular structure files (*n* compounds) in SDF format.
ii. Perform DFT calculations using Gaussian with the 6‐31G(d, p) basis set to obtain IR spectra.
iii. Sort the IR spectra by wavenumber and select the target spectral region (0–4000 cm^−1^ or 0–1700 cm^−1^).
iv. Apply *k*‐means clustering to divide the wavenumber data into 5–20 clusters.
v. Extract the highest intensity or corresponding wavenumber within each cluster and vectorize the results as IntIR or WaveIR descriptors.

## 3 Results and Discussion

### 3.1 Datasets

To evaluate the descriptors derived from IR spectra, we used two datasets: one for direct Pd‐catalyzed arylation reactions reported by Shields et al. [2], and another for SMC reactions reported by Perera et al. [4].

#### 3.1.1 Dataset A: Direct Pd‐Catalyzed Arylation

**SCHEME 1:**
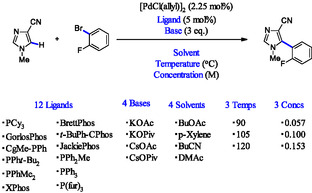
Dataset A: Direct Pd‐catalyzed arylation.

The direct Pd‐catalyzed arylation dataset involves phosphine ligands. It comprises 1,728 reaction conditions generated from combinations of 12 ligands, four bases, four solvents, and three levels of concentration and temperature. Reaction yields are recorded for each condition (Scheme 1, Table S1).

Among these, 494 entries with 0% yield were excluded, resulting in a final dataset of 1,234 entries. Grouping fundamentally different reactions under the same 0% label could distort the relationship between features and yield, biasing the model toward binary classification rather than accurate prediction. Their exclusion enabled more consistent modeling and precise evaluation of predictive performance.

Ligands were encoded using IR‐derived descriptors, while bases and solvents were encoded using OHE. Temperature values were converted from Celsius to Kelvin. In regression analysis, yield was treated as the target variable, with ligand, base, solvent, concentration, and temperature as input features.

#### 3.1.2 Dataset B: Suzuki–Miyaura Coupling

To enable comparison with the direct Pd‐catalyzed arylation dataset, the SMC dataset was preprocessed accordingly. The original dataset includes seven types of substrates 1 (1a–g) and four types of substrates 2 (2a–d), with experimental results recorded for each combination (Scheme S1).

For this study, we selected data corresponding to the 1b–2a combination, which exhibited the highest standard deviation (STD) in yield (Table S2). Additionally, since all ligands in the direct Pd‐catalyzed arylation dataset were monodentate phosphines, we excluded bidentate phosphines, iron‐based ligands, and ligand‐free samples.

**SCHEME 2:**
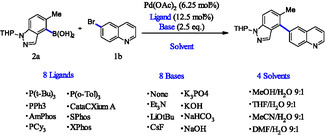
Dataset B: Suzuki–Miyaura coupling.

The final processed dataset consisted of 256 reaction conditions derived from combinations of eight ligands, eight bases, and four solvents (Table S3). As with the direct Pd‐catalyzed arylation dataset, ligands were encoded using IR‐derived descriptors, and bases and solvents were encoded using OHE (Scheme 2).

### 3.2 Evaluation of IR Descriptors

Each ligand was represented by IR‐based descriptors and assigned a unique group ID. To assess generalization performance, we employed the leave‐one‐group‐out (LOGO) cross‐validation method. In each iteration, one ligand group was used as the test set, while the remaining groups were used for training. This process was repeated *n* times, ensuring that all ligands were evaluated as unseen test cases.

This approach enabled rigorous assessment of the model's ability to predict yields for ligands not included in the training data. To evaluate performance on relatively small datasets, the following regression models were tested:

Ordinary least squares (OLS)

Partial least squares (PLS)

Ridge regression (RR)

Least absolute shrinkage and selection operator (LASSO)

Elastic net (EN)

Support vector regression with linear kernel

Support vector regression with Gaussian kernel (SVRG)

Random forests (RF)

Light gradient boosting machine (LGB)

The hyperparameters for each regression model are shown in Table S4. Hyperparameter optimization was performed within the inner cross‐validation of the nested cross‐validation procedure.

Predicted yields were compared with experimental values using the coefficient of determination (*R^2^*) and mean absolute error (MAE) as performance metrics. In addition, to capture general reactivity trends beyond absolute prediction accuracy, rank‐based metric, such as Spearman's rank correlation coefficient (*ρ*), was also employed [24, 25]. These complementary metrics provide insight into the model's ability to reproduce relative ordering of reaction outcomes, which is particularly relevant for condition selection and optimization. The regression method that achieved the highest predictive accuracy on the test data was selected for model construction.

**FIGURE 1:**
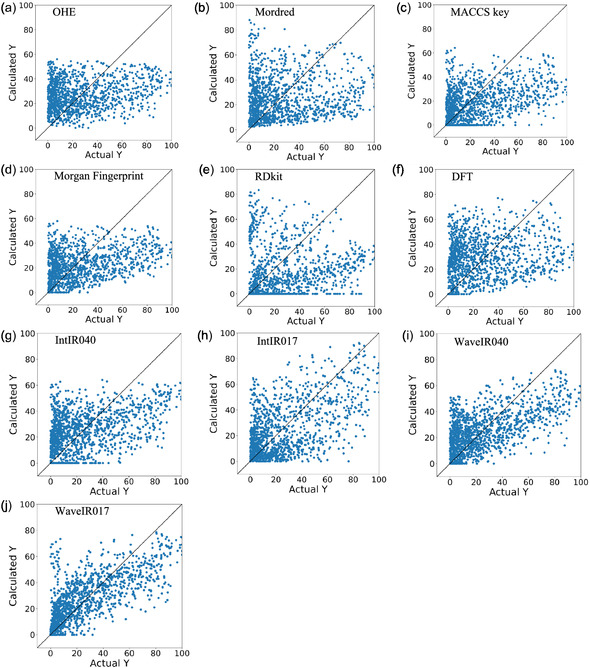
Actual *y* versus estimated *y* for the direct Pd‐catalyzed arylation data. (a) OHE (PLS), (b) Mordred (RF), (c) MACCS key (LASSO), (d) Morgan fingerprint (EN), (e) RDKit (LSVR), (f) DFT (LASSO), (g) IntIR040 (LASSO), (h) IntIR017 (LGB), (i) WaveIR040 (PLS), and (j) WaveIR017 (OLS).

**FIGURE 2:**
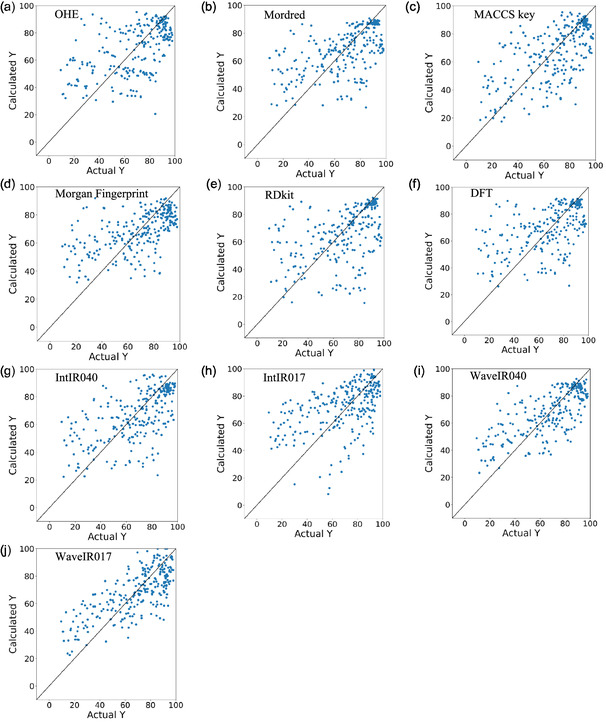
Actual *y* versus estimated *y* for the SMC data. (a) OHE (SVRG), (b) Mordred (RF), (c) MACCS key (RF), (d) Morgan fingerprint (SVRG), (e) RDKit (RF), (f) DFT (RF), (g) IntIR040 (RF), (h) IntIR017(LGB), (i) WaveIR040 (RF), and (j) WaveIR017 (PLS).

For comparison, we also evaluated datasets in which ligands were represented using conventional descriptors: OHE, Mordred, MACCS keys, Morgan fingerprints, RDKit [26], and DFT descriptors. OHE was included as a baseline control, and DFT descriptors were obtained from the KRAKEN descriptor library [27]. Results for the direct Pd‐catalyzed arylation dataset are shown in Table 1, Figure 1, and Table S5–S13, and those for the SMC dataset are shown in Table 2, Figure 2, and Table S14–S22.

**TABLE 1:** *R* ^2^, MAE, and *ρ* for each descriptor in the direct Pd‐catalyzed arylation dataset.

Descriptor	Cluster number	Regression method	*R* ^2^	MAE	*ρ*
OHE	—	PLS	−0.02	20.74	0.17
Mordred	—	RF	−0.45	23.22	0.11
MACCS key	—	LASSO	0.02	19.03	0.37
Morgan fingerprint	—	EN	0.05	19.30	0.29
RDKit	—	LSVR	−0.51	23.23	0.28
DFT	—	LASSO	−0.20	22.13	0.20
IntIR040	20	LASSO	0.11	19.37	0.34
IntIR017	18	LGB	0.22	17.42	0.31
WaveIR040	11	PLS	0.33	16.36	0.50
WaveIR017	10	OLS	0.49	13.35	0.71

**TABLE 2:** *R* ^2^, MAE and *ρ* for each descriptor in the SMC dataset.

Descriptor	Cluster number	Regression method	R^2^	MAE	*ρ*
OHE	—	SVRG	0.23	16.30	0.54
Mordred	—	RF	0.27	15.07	0.59
MACCS key	—	RF	0.36	14.27	0.64
Morgan fingerprint	—	SVRG	0.31	15.04	0.61
RDKit	—	RF	0.07	16.16	0.53
DFT	—	RF	0.25	15.13	0.58
IntIR040	6	RF	0.34	14.48	0.64
IntIR017	5	LGB	0.16	17.04	0.56
WaveIR040	12	RF	0.54	12.26	0.72
WaveIR017	6	PLS	0.54	13.00	0.71

### 3.3 Performance Comparison and Descriptor Analysis

In both the direct Pd‐catalyzed arylation and SMC datasets, wavenumber‐based descriptors derived from IR spectra demonstrated superior predictive performance compared to models using conventional molecular descriptors and structural fingerprints (OHE, Mordred, MACCS, Morgan fingerprint, RDKit, and DFT). Specifically, in the direct Pd‐catalyzed arylation dataset, the WaveIR017 descriptor combined with the OLS model achieved the best results with *R* ^2^ = 0.49, MAE = 13.35, and *ρ* = 0.71. In the SMC dataset, the WaveIR040 descriptor with the RF model yielded the highest performance with *R* ^2^ = 0.54, MAE = 12.26, and *ρ* = 0.72. These results suggest that IR spectra effectively capture both structural and electronic features, and that the descriptors successfully extracted relevant information for yield prediction.

Furthermore, the use of LOGO cross‐validation confirmed the model's ability to generalize to unseen ligands, indicating that IR‐based descriptors can maintain strong predictive performance even in small‐scale datasets.

However, the choice of wavenumber range for IR spectra remains a point of discussion. In the direct Pd‐catalyzed arylation dataset, restricting the input range from 0–4000 to 0–1700 cm^−1^ improved prediction accuracy, whereas in the SMC dataset, both ranges yielded comparable performance. This observation can be explained by the nature of IR spectral regions. 1700–4000 cm^−1^ corresponds to the functional group region, while 0–1700 cm^−1^ represents the fingerprint region. According to Punjabi et al. [28], who studied functional group extraction in chemometrics, the fingerprint region contains rich structural information and enhances classification accuracy of functional groups. Therefore, the 0–1700 cm^−1^ range is considered more suitable for capturing electronic and structural features of molecules. Including the 1700–4000 cm^−1^ region may introduce noise and negatively affect model performance.

In contrast, intensity‐based descriptors showed inferior predictive performance in both datasets. This is likely due to the sensitivity of IR intensity calculations to electron distribution and computational parameters, which can compromise stability. Near‐infrared intensity predictions require anharmonic corrections, which are computationally expensive and unsuitable for large or flexible molecules. Additionally, when using experimental data, intensity values must be corrected for concentration effects based on the Beer–Lambert law, further complicating comparisons across samples. These factors suggest that wavenumber‐based descriptors offer better consistency and are more suitable for ML applications from both theoretical and experimental perspectives.

### 3.4 Analysis of Prediction Deviations

**FIGURE 3:**
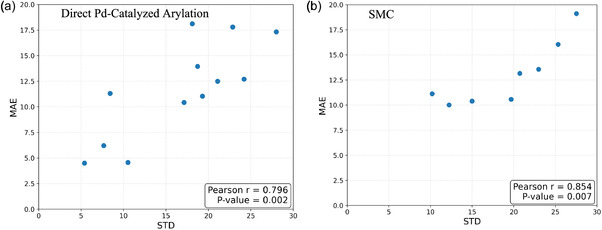
Correlation between the STD of experimental yields and the MAE of prediction results for each ligand group set. (a) Direct Pd‐catalyzed arylation dataset. (b) SMC dataset. STD = Standard deviation; MAE = mean absolute error; SMC = Suzuki–Miyaura coupling.

To investigate factors contributing to discrepancies between predicted and actual yields, we analyzed the results of the WaveIR017 descriptor for both datasets, as shown in Tables 1 and 2. Interestingly, a positive correlation was observed between the STD of actual yields and the MAE of the predictions for each ligand (Figure 3, Table S23–24). This correlation was statistically significant in both datasets (Dataset 1: Pearson *r* = 0.796, *p* = 0.00194; Dataset 2: Pearson *r* = 0.854, *p* = 0.00699), indicating that greater variability in experimental yields is strongly associated with increased prediction error.

**FIGURE 4:**
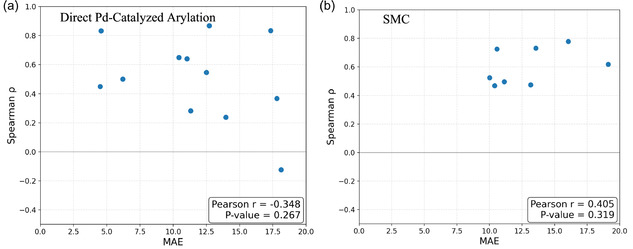
Correlation between the MAE and *ρ* of prediction results for each ligand group set. (a) Direct Pd‐catalyzed arylation dataset. (b) SMC dataset. MAE = Mean absolute error; SMC = Suzuki–Miyaura coupling.

The relationship between MAE and *ρ* was examined. The initial hypothesis was that an increase in MAE would correspond to a decrease in *ρ*, implying that larger prediction errors would lead to poorer ranking accuracy. However, evaluations across two datasets revealed no statistically significant association. Specifically, dataset 1 showed a weak negative correlation (Pearson *r* = −0.348, *p* = 0.267), whereas dataset 2 exhibited a weak positive correlation (Pearson *r* = 0.405, *p* = 0.319). In both cases, the *p*‐values were well above the conventional significance threshold (*α* = 0.05), indicating no clear linear relationship between MAE and ranking accuracy (Figure 4).

These findings suggest that an increase in MAE does not necessarily imply a deterioration in ranking accuracy. In other words, ranking performance may remain relatively stable even when absolute prediction errors are large. This observation is particularly relevant for experimental condition selection, as it highlights that WaveIR017, despite room for improvement in absolute error, retains practical utility for guiding efficient exploration. It is important to note that MAE evaluates absolute prediction error, whereas Spearman's correlation assesses rank agreement; thus, the two metrics do not necessarily correlate. Even when prediction errors are large, accurate ranking can preserve high search efficiency.

Additionally, in this study, reaction parameters other than ligands (such as solvents and bases) were encoded using OHE. Incorporating more expressive descriptors could better capture these features and potentially improve the predictive performance of the model. Future work should focus on redefining the reaction condition space and enhancing feature representation to further improve model accuracy.

## 4 Conclusion

In this study, we designed descriptors based on the IR spectra of ligands and evaluated their applicability to yield prediction models. Using datasets from direct Pd‐catalyzed arylation and SMC reactions, we demonstrated that wavenumber‐based IR descriptors achieved superior predictive performance for unseen ligand data compared to conventional molecular descriptors and structural fingerprints (OHE, Mordred, MACCS, Morgan fingerprint, RDKit, and DFT).

Notably, descriptors restricted to the fingerprint region (0–1700 cm^−1^) more effectively captured the electronic and structural features of molecules, contributing to both improved prediction accuracy and enhanced chemical interpretability. In contrast, intensity‐based descriptors showed inferior performance due to their sensitivity to electron distribution and computational conditions, which can compromise stability. Importantly, wavenumber‐based features proved to be more reliable, as they reflect molecular characteristics while maintaining computational robustness. Even in experimentally acquired spectra, wavenumber information is more consistent across samples, supporting broader applications of IR spectral data in ML‐based chemical analysis.

A statistically significant positive correlation between STD and MAE underscores that greater experimental variability strongly contributes to prediction error. Conversely, no statistically significant relationship was observed between MAE and Spearman's rank correlation (*ρ*) across datasets, indicating that larger prediction errors do not necessarily degrade ranking accuracy. This suggests that the proposed approach retains practical utility for condition prioritization even when absolute errors are high. Improving the representation of other reaction parameters, such as solvents and bases, which were encoded using OHE in this study, remains an important direction for enhancing model performance.

Descriptor design based on IR spectra shows strong potential for generalization, even in small datasets, and offers a promising approach for redefining experimental spaces in reaction optimization. By simultaneously encoding electronic and structural features, IR‐based descriptors are expected to serve as a valuable tool for guiding experimental design and enhancing the interpretability of predictive models.

## Supporting Information

Additional SI can be found online in the Supporting Information section. **Supporting Scheme S1:** Substrates of SMC reaction in dataset B. **Supporting Table S1:** List of compounds in dataset A. **Supporting Table S2:** The STD of yield for each combination of substrates 1 and 2 in dataset B. **Supporting Table S3:** List of compounds in dataset B. **Supporting Table 4**: The list of hyperparameters for regression models. **Supporting Table S5:** MAE with general predictor in dataset A. **Supporting Table S6:** MAE with IntIR040 predictor in dataset A (cluster number 5‐12). **Supporting Table S7:** MAE with IntIR040 predictor in dataset A (cluster number 13−20). **Supporting Table S8:** MAE with IntIR017 predictor in dataset A (cluster number 5–12). **Supporting Table S9:** MAE with IntIR017 predictor in dataset A (cluster number 13−20). **Supporting Table S10:** MAE with WaveIR040 predictor in dataset A (cluster number 5–12). **Supporting Table S11:** MAE with WaveIR040 predictor in dataset A (cluster number 13−20). **Supporting Table S12:** MAE with WaveIR017 predictor in dataset A (cluster number 5–12). **Supporting Table S13:** MAE with WaveIR017 predictor in dataset A (cluster number 13−20). **Supporting Table S14:** MAE with general predictor in dataset B. **Supporting Table S15:** MAE with IntIR040 predictor in dataset B (cluster number 5–12). **Supporting Table S16:** MAE with IntIR040 predictor in dataset B (cluster number 13−20). **Supporting Table S17:** MAE with IntIR017 predictor in dataset B (cluster number 5–12). **Supporting Table S18:** MAE with IntIR017 predictor in dataset B (cluster number 13−20). **Supporting Table S19:** MAE with WaveIR040 predictor in dataset B (cluster number 5–12). **Supporting Table S20:** MAE with WaveIR040 predictor in dataset B (cluster number 13−20). **Supporting Table S21:** MAE with WaveIR017 predictor in dataset B (cluster number 5–12). **Supporting Table S22:** MAE with WaveIR017 predictor in dataset B (cluster number 13−20). **Supporting Table S23:** STD of actual yields, and MAE and *ρ* of the predictions for each ligand in dataset A. **Supporting Table S24:** STD of actual yields, and the MAE and *ρ* of the predictions for each ligand in dataset B.

## Funding

This study was supported by Japan Society for the Promotion of Science (24K08152 and 24K01234).

## Conflicts of Interest

The authors declares no conflicts of interest.

## Supporting information

Supplementary Material

## Data Availability

The data that support the findings of this study are available in the supporting information (SI) of this article.
