# Supplementary material for: Infrared Spectral Descriptors for Reaction Yield Prediction: Toward Redefining Experimental Spaces
Source: Mol Inform. 2026 Feb 12;45(2):e70019. doi: 10.1002/minf.70019 (PMC12899324; doi:10.1002/minf.70019)
Supplement: Supplementary file 1 — Supplementary Material [file MINF-45-e70019-s001.pdf]

SUPPORTING INFORMATION

Infrared Spectral Descriptors for Reaction Yield Prediction: Toward Redefining Experimental Spaces

Yuya Endo, Hiromasa Kaneko*

Department of Applied Chemistry, School of Science and Technology, Meiji University, 1-1-1 Higashi-Mita, Tama-ku, Kawasaki, Kanagawa 214-8571, Japan

*E-mail: hkaneko@meiji.ac.jp

Contents

Table S1. List of compounds in dataset A 3

Scheme S1. Substrates of Suzuki-Miyaura coupling reaction in dataset B 4

Table S2. The standard deviation of yield for each combination of substrates 1 and 2 in dataset B 4

Table S3. List of compounds in dataset B 5

Table S4. The list of hyperparameters for regression models 6

Table S5. MAE with general predictor in dataset A 6

Table S6. MAE with IntIR040 predictor in dataset A (cluster number 5-12) 7

Table S7. MAE with IntIR040 predictor in dataset A (cluster number 13-20) 7

Table S8. MAE with IntIR017 predictor in dataset A (cluster number 5-12) 8

Table S9. MAE with IntIR017 predictor in dataset A (cluster number 13-20) 8

Table S10. MAE with WaveIR040 predictor in dataset A (cluster number 5-12) 9

Table S11. MAE with WaveIR040 predictor in dataset A (cluster number 13-20) 9

Table S12. MAE with WaveIR017 predictor in dataset A (cluster number 5-12) 10

Table S13. MAE with WaveIR017 predictor in dataset A (cluster number 13-20) 10

Table S14. MAE with general predictor in dataset B 11

Table S15. MAE with IntIR040 predictor in dataset B (cluster number 5-12) 11

Table S16. MAE with IntIR040 predictor in dataset B (cluster number 13-20) 12

Table S17. MAE with IntIR017 predictor in dataset B (cluster number 5-12) 12

Table S18. MAE with IntIR017 predictor in dataset B (cluster number 13-20) 13

Table S19. MAE with WaveIR040 predictor in dataset B (cluster number 5-12) 13

Table S20. MAE with WaveIR040 predictor in dataset B (cluster number 13-20) 14

Table S21. MAE with WaveIR017 predictor in dataset B (cluster number 5-12) 14

Table S22. MAE with WaveIR017 predictor in dataset B (cluster number 13-20) 15

Table S23. STD of actual yields, and MAE and ρ of the predictions for each ligand in dataset A 15

Table S24. STD of actual yields, and the MAE and ρ of the predictions for each ligand in dataset B 16

ABBREVIATIONS 16

Table S1. List of compounds in dataset A

Name	CAS No.
PCy_3	2622-14-2
GorlosPhos	1268824-69-6
CgMe-PPh	97739-46-3
PPh*t*-Bu_2	32673-25-9
$PPhMe_2$	672-66-2
XPhos	564483-18-7
BrettPhos	1070663-78-3
t-BuPh-CPhos	1660153-91-2
JackiePhos	1160861-60-8
PPh_2Me	1486-28-8
PPh_3	603-35-0
$P(fur)_3$	5518-52-5
KOAc	127-08-2
KOPiv	19455-23-3
CsOAc	3396-11-0
CsOPiv	20442-70-0
BuOAc	540-88-5
p-Xylene	106-42-3
BuCN	109-74-0
DMAc	127-19-5

Scheme S1. Substrates of Suzuki-Miyaura coupling reaction in dataset B

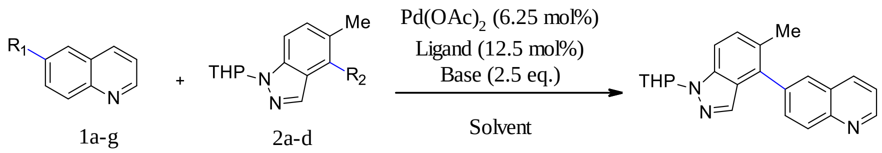

1a-g

- **1a** R_1 = Cl
- **1b** R_1 = Br
- **1c** R_1 = OTf
- **1d** R_1 = I
- **1e** R_1 = $B(OH)_2$
- **1f** R_1 = Bpin
- **1g** R_1 = BF_3K

2a-d

- **2a** R_2 = $B(OH)_2$
- **2b** R_2 = Bpin
- **2c** R_2 = BF_3K
- **2d** R_2 = Br

Table S2. The standard deviation of yield for each combination of substrates 1 and 2 in dataset B

Substrate	**2a** R_2 = $B(OH)_2$	**2b** R_2 = Bpin	**2c** R_2 = BF_3K	**2d** R_2 = Br
**1a** R_1 = Cl	24.91	19.99	6.08	-
**1b** R_1 = Br	**30.34**	27.25	16.14	-
**1c** R_1 = OTf	26.22	25.43	16.72	-
**1d** R_1 = I	18.92	20.46	23.57	-
**1e** R_1 = $B(OH)_2$	-	-	-	21.52
**1f** R_1 = Bpin	-	-	-	23.57
**1g** R_1 = BF_3K	-	-	-	22.36

Table S3. List of compounds in dataset B

Name	CAS No.
P(*t*-Bu)$_3$	13716-12-6
PPh$_3$	603-35-0
AmPhos	932710-63-9
PCy$_3$	2622-14-2
P(o-Tol)$_3$	6163-58-2
CataCXium A	321921-71-5
SPhos	657408-07-6
XPhos	564483-18-7
Et$_3$N	121-44-8
LiO*t*Bu	1907-33-1
CsF	13400-13-0
K_3PO_4	115281-28-2
KOH	1310-58-3
$NaHCO_3$	144-55-8
NaOH	1310-73-2
MeOH	67-56-1
THF	109-99-9
MeCN	75-05-8
DMF	68-12-2
H_2O	7732-18-5

Table S4. The list of hyperparameters for regression models

Model	Hyperparameters
OLS	None
PLS	n_components
Ridge	α
LASSO	α
Elastic Net	α, l1_ratio
SVR (Linear)	C, ε
SVR (Gaussian)	C, ε, γ
Random Forest	n_estimators, max_features
LGB	boosting_type, learning_rate, num_leaves

Table S5. MAE with general predictor in dataset A

Regression model	OHE	Mordred	MACCS key	Morgan Fingerprint	RDKit	DFT
OLS	47.41	45.44	55.12	53.19	42.12	37.13
PLS	20.74	28.38	19.84	19.37	23.57	24.11
RR	20.84	28.24	19.80	19.34	23.70	24.09
LASSO	20.56	32.68	19.03	21.33	31.64	22.13
EN	20.82	30.81	19.19	19.30	24.03	24.22
SVRL	20.86	28.30	19.71	19.40	23.24	24.34
SVRG	22.62	25.18	22.95	22.10	23.64	23.58
RF	22.99	23.22	23.79	22.83	24.71	23.43
LGB	24.11	27.80	20.55	27.38	28.42	29.16

Table S6. MAE with IntIR040 predictor in dataset A (cluster number 5-12)

Regression	Cluster number							
model	5	6	7	8	9	10	11	12
OLS	40.10	39.85	38.64	49.60	62.33	56.40	50.72	61.92
PLS	28.17	27.87	27.00	28.50	28.80	27.96	28.80	21.73
RR	33.83	36.45	37.03	45.58	62.29	27.93	55.85	21.67
LASSO	34.22	36.15	37.30	43.39	57.97	32.82	51.33	22.62
EN	33.74	35.72	38.54	42.49	58.08	31.68	51.32	20.07
SVRL	35.16	37.07	37.92	49.64	61.19	27.23	55.76	22.10
SVRG	29.76	32.10	29.58	27.02	26.32	23.92	30.55	26.19
RF	25.44	23.35	24.04	21.26	20.40	21.47	22.43	22.36
LGB	27.24	26.61	25.83	21.75	20.83	21.34	26.49	24.55

Table S7. MAE with IntIR040 predictor in dataset A (cluster number 13-20)

Regression	Cluster number							
model	13	14	15	16	17	18	19	20
OLS	61.65	36.97	45.54	44.75	47.65	50.72	51.72	55.71
PLS	29.66	25.39	28.04	34.39	23.26	28.62	27.48	21.86
RR	34.47	27.34	32.02	36.69	23.20	34.04	27.52	21.38
LASSO	31.19	25.91	22.17	34.69	31.28	31.82	24.09	19.37
EN	28.94	24.49	27.21	43.44	35.14	29.24	25.39	20.53
SVRL	34.19	28.46	28.71	36.50	24.39	33.39	28.99	22.02
SVRG	23.19	26.53	25.46	24.92	28.19	27.42	24.91	22.03
RF	20.20	21.86	22.58	22.91	19.22	20.03	24.52	22.28
LGB	19.00	23.95	22.20	26.46	22.67	22.17	27.14	22.90

Table S8. MAE with IntIR017 predictor in dataset A (cluster number 5-12)

Regression	Cluster number							
model	5	6	7	8	9	10	11	12
OLS	27.97	25.65	21.16	41.93	40.04	26.44	64.99	58.54
PLS	26.33	25.05	21.21	26.43	26.30	26.37	23.92	26.51
RR	28.22	26.08	21.79	41.31	40.25	26.27	24.91	25.87
LASSO	28.01	26.60	21.32	42.03	40.21	26.20	25.85	28.55
EN	28.16	26.26	21.16	41.59	40.01	26.37	26.08	28.67
SVRL	28.33	27.87	21.81	41.06	40.60	27.28	25.06	28.82
SVRG	43.47	34.20	30.73	30.20	33.15	26.65	27.62	29.47
RF	28.54	22.16	24.94	25.23	26.37	25.07	22.90	25.41
LGB	29.43	21.70	25.43	27.18	28.99	30.07	23.61	30.02

Table S9. MAE with IntIR017 predictor in dataset A (cluster number 13-20)

Regression	Cluster number							
model	13	14	15	16	17	18	19	20
OLS	49.98	53.18	41.06	56.54	49.94	43.49	44.40	45.10
PLS	24.59	24.51	23.55	23.08	25.86	21.70	25.78	25.70
RR	31.41	24.22	24.50	23.11	28.27	22.11	26.37	36.87
LASSO	36.32	21.94	26.89	21.09	31.35	28.31	26.21	42.49
EN	38.23	23.91	28.98	22.85	31.04	26.33	28.44	42.13
SVRL	33.07	27.52	24.32	22.03	28.83	23.26	27.37	37.02
SVRG	23.82	24.99	27.34	25.61	25.72	24.06	24.75	24.73
RF	21.09	21.42	24.22	21.63	23.01	21.72	23.04	20.32
LGB	21.34	21.99	25.43	19.15	24.17	20.73	20.05	18.94

Table S10. MAE with WaveIR040 predictor in dataset A (cluster number 5-12)

Regression	Cluster number							
model	5	6	7	8	9	10	11	12
OLS	26.49	23.28	23.35	24.98	36.35	42.85	46.22	53.18
PLS	19.68	21.00	17.84	17.66	18.33	21.54	16.36	17.18
RR	24.56	22.63	23.21	25.69	36.26	21.84	23.18	31.50
LASSO	25.47	23.07	23.34	23.21	35.88	23.18	28.57	29.07
EN	24.60	22.69	23.50	24.76	36.19	21.65	29.18	28.92
SVRL	24.04	23.71	23.30	26.59	33.66	23.40	24.06	30.37
SVRG	29.99	24.45	23.03	17.67	18.99	21.97	18.69	22.37
RF	22.92	22.49	18.51	17.29	21.18	22.84	21.53	22.87
LGB	24.27	25.45	19.02	18.55	21.37	24.43	22.22	21.67

Table S11. MAE with WaveIR040 predictor in dataset A (cluster number 13-20)

Regression	Cluster number							
model	13	14	15	16	17	18	19	20
OLS	57.40	41.14	52.57	54.13	44.06	51.53	40.00	59.03
PLS	18.85	17.61	17.66	20.81	26.54	20.43	21.35	18.98
RR	38.69	32.94	21.54	21.36	26.37	21.75	23.11	22.06
LASSO	37.71	33.94	22.64	23.22	24.27	20.24	20.59	26.51
EN	38.58	31.84	21.75	24.29	21.13	22.44	22.87	23.85
SVRL	38.86	31.62	21.85	21.11	25.72	21.60	23.39	21.28
SVRG	18.55	21.75	17.98	21.71	23.70	23.40	21.51	21.42
RF	20.69	21.96	22.44	20.58	27.44	21.68	20.35	23.54
LGB	21.11	25.67	21.06	23.25	28.73	24.03	23.04	23.46

Table S12. MAE with WaveIR017 predictor in dataset A (cluster number 5-12)

Regression	Cluster number							
model	5	6	7	8	9	10	11	12
OLS	26.93	18.89	22.14	31.22	25.01	13.35	53.87	57.99
PLS	22.30	16.63	18.44	21.04	25.87	13.47	28.10	15.91
RR	25.66	17.43	21.85	29.35	25.13	13.57	28.87	22.24
LASSO	26.51	17.97	21.94	29.58	25.28	14.79	28.56	24.94
EN	25.90	17.29	22.00	28.77	25.63	14.60	30.25	25.99
SVRL	23.97	16.63	21.88	28.94	23.97	14.48	28.65	20.96
SVRG	21.14	18.94	21.65	27.37	19.00	23.95	26.21	18.93
RF	22.28	21.39	20.87	21.70	20.30	22.89	19.91	24.14
LGB	24.24	21.78	22.56	26.41	26.90	28.11	21.65	27.75

Table S13. MAE with WaveIR017 predictor in dataset A (cluster number 13-20)

Regression	Cluster number							
model	13	14	15	16	17	18	19	20
OLS	42.58	48.10	33.63	58.35	52.86	61.18	48.98	50.20
PLS	19.20	19.76	21.17	19.19	20.51	17.99	17.38	17.56
RR	22.16	19.63	41.61	21.69	39.26	19.76	17.83	17.55
LASSO	22.22	18.50	38.45	22.45	40.52	20.45	15.80	19.37
EN	24.75	19.90	36.88	26.25	42.60	22.98	17.88	17.65
SVRL	21.60	20.04	41.63	21.45	38.23	19.02	18.06	17.39
SVRG	19.39	21.44	24.57	21.12	24.12	17.61	18.34	19.05
RF	19.26	21.56	22.69	22.46	24.49	22.09	21.62	21.15
LGB	21.02	25.29	25.97	24.51	29.97	29.17	25.31	23.43

Table S14. MAE with general predictor in dataset B

Regression model	OHE	Mordred	MACCS key	Morgan Fingerprint	RDKit	DFT
OLS	44.75	53.31	50.76	44.89	46.96	53.87
PLS	17.38	25.93	17.92	16.58	23.43	22.63
RR	17.51	23.47	18.05	16.64	26.68	22.65
LASSO	17.97	31.37	17.74	16.53	25.24	23.44
EN	17.92	29.90	16.89	17.32	22.56	24.33
SVRL	17.73	23.52	19.16	16.81	28.53	23.05
SVRG	16.31	21.07	16.22	15.03	20.63	19.65
RF	16.35	15.07	14.27	14.82	16.16	15.13
LGB	17.42	19.36	17.45	15.07	20.22	15.92

Table S15. MAE with IntIR040 predictor in dataset B (cluster number 5-12)

Regression	Cluster number							
model	5	6	7	8	9	10	11	12
OLS	17.73	55.95	46.41	56.16	47.10	47.10	62.45	47.38
PLS	18.72	20.33	18.96	21.39	26.92	24.02	22.13	30.56
RR	21.93	20.30	28.03	30.74	30.80	29.73	27.64	30.36
LASSO	23.66	22.60	25.93	26.15	35.85	26.56	29.53	38.09
EN	23.09	20.58	25.65	25.00	33.35	26.99	30.22	33.33
SVRL	29.73	36.99	30.70	29.91	32.13	20.42	25.12	19.75
SVRG	19.66	19.11	22.45	29.36	22.57	18.67	22.01	19.36
RF	17.57	14.48	21.44	15.87	19.24	18.93	17.24	18.72
LGB	16.98	14.84	21.08	17.35	20.89	20.63	18.86	20.14

Table S16. MAE with IntIR040 predictor in dataset B (cluster number 13-20)

Regression	Cluster number							
model	13	14	15	16	17	18	19	20
OLS	53.87	36.17	47.09	46.97	45.30	68.33	51.03	45.15
PLS	26.81	23.73	24.92	25.49	26.73	18.60	26.34	19.23
RR	28.02	31.21	27.46	27.32	29.42	19.08	25.79	18.96
LASSO	23.66	22.60	25.93	26.15	35.85	26.56	29.53	38.09
EN	27.94	28.98	31.05	25.08	29.68	17.03	26.58	21.13
SVRL	29.73	36.99	30.70	29.91	32.13	20.42	25.12	19.75
SVRG	20.19	22.77	21.16	18.33	21.10	18.99	19.63	16.76
RF	18.09	17.70	18.70	20.09	17.81	17.97	19.29	18.27
LGB	16.98	14.84	21.08	17.35	20.89	20.63	18.86	20.14

Table S17. MAE with IntIR017 predictor in dataset B (cluster number 5-12)

Regression	Cluster number							
model	5	6	7	8	9	10	11	12
OLS	26.20	33.71	32.23	31.91	33.84	30.34	48.00	44.24
PLS	24.82	28.13	25.14	26.43	27.93	20.29	26.32	22.19
RR	26.21	34.06	31.58	32.03	33.50	19.95	33.17	22.13
LASSO	25.02	34.28	31.27	32.03	24.08	22.77	28.85	23.88
EN	26.44	33.79	32.08	32.02	25.50	23.47	28.48	25.17
SVRL	26.74	33.90	31.14	31.68	24.61	18.02	32.87	21.11
SVRG	28.17	26.13	29.52	32.33	30.11	25.71	25.54	27.64
RF	22.67	20.47	25.00	24.62	26.09	24.34	20.77	25.20
LGB	25.49	25.02	28.56	27.28	30.14	30.26	19.82	25.85

Table S18. MAE with IntIR017 predictor in dataset B (cluster number 13-20)

Regression	Cluster number							
model	13	14	15	16	17	18	19	20
OLS	49.23	47.01	48.68	58.75	41.25	49.96	44.04	52.45
PLS	21.55	21.16	24.99	25.19	23.14	23.96	25.93	24.75
RR	21.56	21.20	24.75	25.32	23.06	23.59	25.88	24.94
LASSO	28.06	22.34	25.22	27.45	22.24	27.13	29.47	29.57
EN	29.35	27.65	26.82	28.15	19.37	22.56	26.77	25.71
SVRL	22.51	21.46	23.00	25.44	22.46	23.07	25.59	24.60
SVRG	27.93	26.16	26.77	25.76	23.27	23.75	23.64	24.81
RF	21.98	21.10	21.86	21.09	19.61	19.61	22.61	22.53
LGB	19.81	18.61	22.61	19.87	21.44	17.43	24.68	23.18

Table S19. MAE with WaveIR040 predictor in dataset B (cluster number 5-12)

Regression	Cluster number							
model	5	6	7	8	9	10	11	12
OLS	51.58	49.79	42.52	34.91	56.78	55.27	40.53	58.79
PLS	22.92	18.52	13.86	14.93	18.19	14.75	17.65	15.32
RR	50.75	19.18	14.15	17.63	18.15	14.97	18.62	15.66
LASSO	49.54	19.06	14.69	15.62	19.60	14.84	20.62	15.94
EN	49.04	18.93	14.34	15.63	19.45	14.79	21.29	16.00
SVRL	47.80	19.41	14.13	22.70	18.53	14.94	19.22	16.01
SVRG	35.45	17.12	14.74	15.49	18.29	13.37	17.89	15.47
RF	18.17	14.74	15.06	15.05	18.98	12.99	22.44	12.26
LGB	18.81	15.31	13.95	14.59	17.66	14.41	23.24	13.65

Table S20. MAE with WaveIR040 predictor in dataset B (cluster number 13-20)

Regression	Cluster number							
model	13	14	15	16	17	18	19	20
OLS	49.09	48.68	37.96	46.54	47.58	61.62	52.98	47.38
PLS	16.10	16.44	16.56	18.00	26.22	18.55	23.91	21.18
RR	16.15	16.79	16.70	18.38	27.68	18.68	23.53	21.03
LASSO	15.95	18.38	16.70	21.02	31.03	19.48	26.01	22.52
EN	15.35	18.26	15.94	19.94	30.70	17.84	26.18	22.66
SVRL	15.77	16.61	16.95	19.14	28.64	18.76	23.76	21.18
SVRG	14.51	16.08	15.89	16.49	19.81	16.54	18.09	16.61
RF	14.50	15.09	15.48	16.75	23.85	15.86	22.56	15.77
LGB	16.57	15.67	16.28	21.21	25.97	19.32	20.23	16.53

Table S21. MAE with WaveIR017 predictor in dataset B (cluster number 5-12)

Regression	Cluster number							
model	5	6	7	8	9	10	11	12
OLS	44.29	31.67	51.04	49.10	49.10	40.53	38.85	57.82
PLS	21.35	13.00	15.88	15.57	17.02	17.63	24.94	24.74
RR	37.47	13.59	14.75	15.76	17.15	17.83	24.70	27.73
LASSO	36.83	14.08	14.91	14.03	15.14	18.26	28.20	26.76
EN	36.59	14.05	14.92	14.10	14.99	18.44	27.96	26.11
SVRL	42.24	13.29	22.91	16.03	17.33	18.17	25.08	29.01
SVRG	25.03	12.69	14.18	15.22	16.20	19.70	20.07	22.46
RF	14.36	14.78	13.90	15.63	12.91	22.90	19.46	22.02
LGB	17.31	13.90	15.40	15.43	14.86	22.38	21.53	21.96

Table S22. MAE with WaveIR017 predictor in dataset B (cluster number 13-20)

Regression model	Cluster number 13	14	15	16	17	18	19	20
OLS	46.41	52.98	61.76	59.61	44.45	50.35	42.74	44.87
PLS	20.49	21.64	22.69	25.22	20.07	17.44	19.71	26.65
RR	20.66	21.46	22.44	24.89	20.51	17.38	19.57	25.11
LASSO	18.81	19.71	21.91	21.49	16.61	21.23	21.15	22.32
EN	19.12	19.79	21.40	21.24	17.12	20.85	21.45	23.12
SVRL	21.10	21.39	22.27	25.41	20.83	17.76	19.78	25.89
SVRG	18.07	16.88	18.57	20.26	17.92	17.43	17.78	19.79
RF	15.35	16.96	16.85	17.07	18.96	14.21	22.29	19.07
LGB	18.72	16.90	16.20	17.33	18.91	17.22	22.53	19.12

Table S23. STD of actual yields, and MAE and ρ of the predictions for each ligand in dataset A

Ligand	standard deviation of actual yields	MAE of the predictions	ρ of the predictions
PCy_3	19.299	4.563	0.833
GorlosPhos	17.134	10.424	0.649
CgMe-PPh	27.997	17.339	0.835
PPh*t*-Bu_2	8.420	11.316	0.282
$PPhMe_2$	5.397	4.496	0.450
XPhos	22.868	17.806	0.367
BrettPhos	18.098	18.118	-0.123
t-BuPh-CPhos	18.716	13.956	0.237
JackiePhos	21.082	12.500	0.546
PPh_2Me	7.667	6.213	0.501
PPh_3	24.201	12.706	0.868
P(fur)$_3$	19.299	4.563	0.833

Table S24. STD of actual yields, and the MAE and ρ of the predictions for each ligand in dataset B

Ligand	standard deviation of actual yields	MAE of the predictions	ρ of the predictions
P(*t*-Bu)$_3$	20.723	13.163	0.474
PPh$_3$	10.195	11.131	0.497
AmPhos	14.990	10.392	0.468
PCy$_3$	19.689	10.570	0.725
P(o-Tol)$_3$	27.522	19.117	0.618
CataCXium A	12.228	10.018	0.525
SPhos	22.978	13.569	0.731
XPhos	25.328	16.049	0.778

ABBREVIATIONS

IR spectra; infrared spectra

HTE; high-throughput experimentation

ML; machine learning

DL; deep learning

DFT; density functional theory

SMC; Suzuki–Miyaura coupling

OHE; one-hot encoding

IntIR; Intensity-based IR descriptor

WaveIR; Wavenumber-based IR descriptor

STD; standard deviation

LOGO; Leave-One-Group-Out

OLS; Ordinary Least Squares

PLS; Partial Least Squares

RR; Ridge Regression

LASSO; Least Absolute Shrinkage and Selection Operator

EN; Elastic Net

SVRL; Support Vector Regression with Linear Kernel

SVRG; Support Vector Regression with Gaussian Kernel

RF; Random Forests

LGB; Light Gradient Boosting Machine

R^2; the coefficient of determination

MAE; mean absolute error

ρ; Spearman's rank correlation coefficient
